# The Influence of Different Prosodic Cues on Word Segmentation

**DOI:** 10.3389/fpsyg.2021.622042

**Published:** 2021-03-16

**Authors:** Theresa Matzinger, Nikolaus Ritt, W. Tecumseh Fitch

**Affiliations:** ^1^Department of English, University of Vienna, Vienna, Austria; ^2^Department of Behavioral and Cognitive Biology, University of Vienna, Vienna, Austria; ^3^Cognitive Science Hub, University of Vienna, Vienna, Austria

**Keywords:** language learning, speech segmentation, prosody, statistical cues, word stress, pauses

## Abstract

A prerequisite for spoken language learning is segmenting continuous speech into words. Amongst many possible cues to identify word boundaries, listeners can use both transitional probabilities between syllables and various prosodic cues. However, the relative importance of these cues remains unclear, and previous experiments have not directly compared the effects of contrasting multiple prosodic cues. We used artificial language learning experiments, where native German speaking participants extracted meaningless trisyllabic “words” from a continuous speech stream, to evaluate these factors. We compared a baseline condition (statistical cues only) to five test conditions, in which word-final syllables were either (a) followed by a pause, (b) lengthened, (c) shortened, (d) changed to a lower pitch, or (e) changed to a higher pitch. To evaluate robustness and generality we used three tasks varying in difficulty. Overall, pauses and final lengthening were perceived as converging with the statistical cues and facilitated speech segmentation, with pauses helping most. Final-syllable shortening hindered baseline speech segmentation, indicating that when cues conflict, prosodic cues can override statistical cues. Surprisingly, pitch cues had little effect, suggesting that duration may be more relevant for speech segmentation than pitch in our study context. We discuss our findings with regard to the contribution to speech segmentation of language-universal boundary cues vs. language-specific stress patterns.

## Introduction

### The Speech Segmentation Problem

When people begin acquiring a new language, a particular challenge is the segmentation of fluent speech into words. This task is especially difficult because continuous speech lacks directly accessible cues to word boundaries. Prominent acoustic cues, such as pauses, are rare and occur only inconsistently (Cole et al., 1980; Saffran et al., 1996a; Cutler et al., 1997; Johnson, 2008). This initial speech segmentation problem is most acute for infants learning their first language but is also daunting for second language learners. For adults, the challenge is particularly apparent when they try to identify discrete words in an unfamiliar foreign language (Johnson and Jusczyk, 2001; Endress and Hauser, 2010; Erickson and Thiessen, 2015). Nonetheless, language learners eventually master the speech segmentation problem with ease.

### Experimental Paradigm and Study Rationale

**Figure 1:**
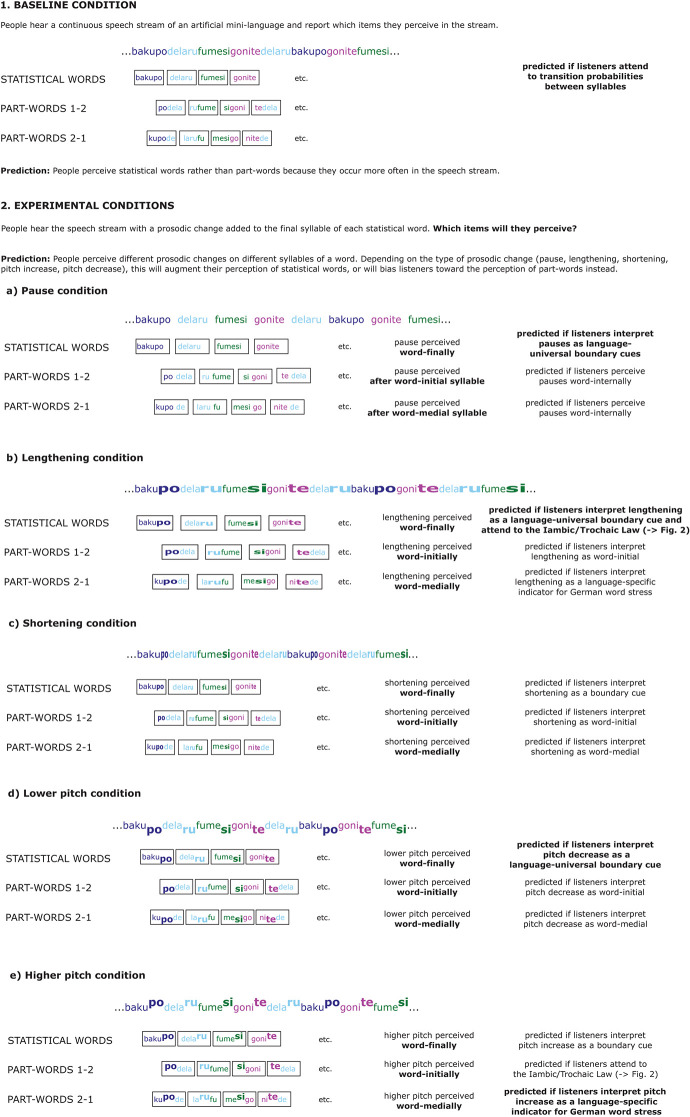
Overview of the study design and predictions. “Part-words 1–2” are created from the final syllable of a statistical word and the initial and medial syllable of the following statistical word. “Part-words 2–1” are created from the medial and final syllable of a statistical word and the initial syllable of the following statistical word. If prosodic cues converge with the statistical cues, participants will perceive the “statistical words.” If prosodic cues conflict with the statistical cues, participants will be biased toward perceiving part-words. The right column contains the most important predictions. Predictions that are derived from previous studies and are therefore most likely to be borne out (for a more detailed discussion, see main text) are highlighted in bold. Predictions that are not informed by evidence-based language-universal and language-specific considerations or are less likely to be borne out are displayed in normal font.

The mechanisms and cues that potentially help language learners extract words from continuous speech have been the subject of a large body of previous research on both infants and adults (e.g., Saffran et al., 1996a,b, 1999; Aslin et al., 1998; Johnson and Jusczyk, 2001; Johnson, 2008, 2012; Johnson and Seidl, 2009; Tyler and Cutler, 2009; Johnson and Tyler, 2010; Hay and Saffran, 2012; Frost et al., 2017). Most of this research used the well-established “artificial language learning” paradigm (Saffran et al., 1996a), which models natural language learning. In this paradigm, listeners are exposed for several minutes to a continuous speech stream of nonsense speech, generated by concatenating invented trisyllabic pseudo-words in a random order. Participants are subsequently tested on the recognition of the intended pseudo-words, as opposed to “part-words”: syllable sequences that occurred due to the juxtaposition of two pseudo-words, which have lower transitional probabilities. For example, listeners might hear the nonsense speech stream …*bakupodelarufumesigonitedelarubakupogonitefumesi…* and infer the recurring trisyllables *bakupo, delaru,fumesi* and *gonite* as acceptable pseudo-words, while rejecting the part-words *kupode, podela* or similar items because these syllables occur in sequence less frequently (e.g., Saffran et al., 1996a; Figure 1: 1. Baseline condition). We will refer to these transitional probabilities between syllables as “statistical cues” and the “words,” i.e., the group of three syllables with the highest internal transitions probabilities (*bakupo, delaru, fumesi*, and *gonite*, in Figure 1) as “statistical words” hereafter.

In this study, we adopted the general design above, but added additional acoustic cues to the nonsense speech stream to investigate how such changes influence listeners' speech segmentation. For simplicity, our study focused on the investigation of prosodic cues on word-final syllables. Thus, durational changes and pitch changes were always implemented on the final syllable of the trisyllabic statistical pseudo-words. Our main aim was to investigate how various prosodic cues such as pauses between statistical words, word-final lengthening, word-final shortening, word-final pitch decrease, and word-final pitch increase influenced which three-syllable groupings German speaking participants segmented from the speech stream as “words” (Figure 1: 2. Experimental conditions). Our second aim was to test how potential language-universal cognitive predispositions and/or language-specific word stress patterns typical of the listeners' native languages influence speech segmentation in an experimental setting (Tyler and Cutler, 2009; Frost et al., 2017; Ordin et al., 2017). We tested German speaking participants because German word stress patterns (most trisyllabic German words are stressed on word-*medial* syllables; Féry, 1998) contrast nicely with language-universal prosodic boundary cues on word-*final* syllables (e.g., phrase-final lengthening; e.g., Fletcher, 2010). If listeners attend to language-universal prosodic boundary cues, adding such cues to the last syllable of a three-syllable statistical word should be perceived as converging with the statistical cues and therefore should facilitate participants' speech segmentation performance (“cue convergence”). In contrast, if listeners interpret such cues as German stress cues, i.e., if they interpret them as occurring word-medially, the prosodic cues would indicate different word boundaries than the boundaries indicated by the statistical cues. Therefore, in this scenario, adding such prosodic cues to the last syllable of a three-syllable statistical word should be perceived as conflicting with the statistical cues. In this case, prosodic cues would hinder speech segmentation based on statistical cues, or even lead to different segmentation patterns than those expected from attending to transition probabilities alone (“cue conflict”). Thus, our paradigm not only compared different prosodic cues, but also helps to disentangle whether adult participants tend to use language-universal or language-specific prosodic cues during speech segmentation. We will explain the study background and our hypotheses in more detail below; see Figure 1 for an overview.

### Speech Segmentation Strategies and Cue Types

Previous research provided abundant evidence that language learners can draw on multiple sources of information for word segmentation (Johnson and Jusczyk, 2001; Mattys et al., 2005; Filippi et al., 2014; Mitchel and Weiss, 2014; Morrill et al., 2015; Johnson, 2016; Sohail and Johnson, 2016), among which “statistical cues” (i.e., transitional probabilities between syllables) and prosodic cues are very prominent.

Using statistical cues present in the speech stream is a very basic language-universal speech segmentation strategy. This strategy is based on tracking transitional probabilities between syllables, which represent the statistical likelihood that one syllable directly follows another (e.g., Saffran et al., 1996a; Aslin et al., 1998; Romberg and Saffran, 2010; Johnson, 2016). Syllables that co-occur frequently are likely to belong to the same word, whereas syllables that co-occur rarely usually span word boundaries (Hayes and Clark, 1970; Swingley, 2005; Johnson and Seidl, 2009; Hay and Saffran, 2012). For example, in the sound sequence “*principal component*,” the transitional probabilities from *prin* to *ci* to *pal* are higher than from *pal* to *com* because *prin, ci* and *pal* also co-occur in other sequences including the word *principal*, such as *principal investigator, principal purpose* or *principal reasons*, whereas *pal* and *com* are only rarely found in immediate succession (frequencies in the Corpus of Contemporary American English: *prin-ci*: 114,277 occurrences, *ci-pal*: 57,520 occurrences, *pal-com*: 1,065 occurrences; Davies 2008). Cross-linguistically, listeners are able to track these statistical relationships, and use them to infer which sound sequences constitute words (Saffran et al., 1996a; Aslin et al., 1998). Still, considerable evidence suggests that statistical cues, while powerful, are not the only information that listeners use to segment speech into words (Morgan and Saffran, 1995; Johnson and Jusczyk, 2001; but also: Thiessen and Saffran, 2003; Johnson and Seidl, 2009; Endress and Hauser, 2010; Johnson and Tyler, 2010; Johnson et al., 2014).

Prosodic cues linked to word stress or word boundaries can provide important additions to statistical cues, and typically enhance speech segmentation performance in infants (e.g., Morgan and Saffran, 1995; Mattys et al., 1999; Johnson and Jusczyk, 2001; Thiessen and Saffran, 2003; Seidl, 2007; Johnson and Seidl, 2009) and adults (e.g., Cutler, 1991; Saffran et al., 1996b; Endress and Mehler, 2009; Endress and Hauser, 2010; Frost et al., 2017). Furthermore, phrasal prosody (e.g., Christophe et al., 2004; Gout et al., 2004; Shukla et al., 2007; Mueller et al., 2010; Langus et al., 2012) and speech pauses (e.g., Johnson et al., 2014; Sohail and Johnson, 2016) facilitate speech segmentation. Compared to statistical cues, which require computations over large sets of syllables, prosodic cues can be extracted relatively directly from the immediate acoustic stimulus (Christophe et al., 2004; Gout et al., 2004; Johnson and Seidl, 2009; Hay and Saffran, 2012; Erickson and Thiessen, 2015), making it reasonable that language learners, especially infants, use them to help solve the speech segmentation problem.

Crucially, prosodic cues can manifest in multiple independent acoustic correlates such as changes in syllable duration, pitch, or loudness, and different acoustic correlates can have different separable effects on speech segmentation (Hay and Saffran, 2012; Ordin and Nespor, 2013). Many previous studies used a combination of different acoustic correlates, but did not determine which prosodic cues were most relevant for word segmentation (Johnson and Jusczyk, 2001; Thiessen and Saffran, 2003, 2007; Johnson and Seidl, 2009). Multiple studies have examined the role of individual cues, suggesting that lengthening serves as a language-universal signal for word-finality (Tyler and Cutler, 2009; Hay and Saffran, 2012; Kim et al., 2012; Frost et al., 2017; but also: White et al., 2020), and that pitch increase is a signal for word stress and is therefore processed differently by speakers of different languages (Morgan and Saffran, 1995; for infants see e.g., Johnson and Jusczyk, 2001; Tyler and Cutler, 2009; Ordin and Nespor, 2016). However, direct comparisons of the roles of different prosodic cues for word segmentation are scarce (e.g., Tyler and Cutler, 2009). To our knowledge, cue changes that contrast in their direction (such as lengthening vs. shortening, or pitch increase vs. decrease) have not been investigated in direct comparison before.

Also, in artificial language learning experiments, prosodic cues that are linked to word stress or word boundaries should only facilitate speech segmentation compared to a statistical baseline if listeners perceive the prosodic cues as converging with the statistical cues defined by the transition probabilities between syllables in the speech stream. For example, in our experiment, if listeners interpret lengthening as a signal for word-finality and perceive it as occurring in word-final position, lengthening should facilitate speech segmentation, since in our experiment, lengthening was always implemented on the final syllable of statistical words. In contrast, if listeners interpret lengthening as a signal for word-initial or word-medial position, listeners should interpret lengthening of the last syllable of statistical words in our experiments as a conflicting cue. In such a case, where prosodic cues conflict with the available statistical cues, prosodic cues could potentially impair speech segmentation relative to statistical cues alone, or even override them and lead to different segmentation patterns (Johnson and Jusczyk, 2001; Thiessen and Saffran, 2003; Johnson and Seidl, 2009; Ordin and Nespor, 2013). Hereafter, we will follow the convention of previous speech segmentation studies (e.g., Frost et al., 2017; Ordin et al., 2017) by defining segmentation that is based on statistical words (potentially enhanced by converging prosodic cues) as the “correct” segmentation. In contrast, if listeners based their segmentation decisions on prosodic cues that conflict with statistical cues, e.g., because they applied a segmentation strategy based on German-specific word-stress patterns, this will be defined in our analyses as “impaired” or “incorrect” segmentation relative to the statistical word baseline. Obviously, such segmentation strategies can also lead to the consistent extraction of items from the speech stream in experimental settings, and there is no intrinsic right or wrong answer in experiments using pseudo-words, but the items resulting from such segmentation strategies clearly differ from the words based on statistical cues alone, which we will henceforth term “correct.” Also, note that our use of statistical cues as a baseline is a product of our experimental design and analysis, and we use the terms “baseline” and “correct” for convenience in describing our results. We clearly do not intend to suggest that statistical cues are somehow primary or “correct” in real-world speech segmentation (and indeed we suspect that prosodic cues might often be dominant): the relative strength of these factors is precisely what our experiments set out to test.

### Choice of Prosodic Cues in Our Study

Although speech segmentation has been widely investigated, it remains unclear which specific acoustic correlates of prosody, such as changes in syllable duration or pitch, are most relevant for speech segmentation. Therefore, the main aim of the current study was to investigate the relative contribution that different acoustic manifestations of prosody make toward speech segmentation in adults. Our study focused on five different prosodic changes in three different acoustic cue categories (Figure 1: 2. Experimental conditions). These were durational cues: (a) syllable lengthening and (b) syllable shortening; voice fundamental frequency or “pitch” cues: (c) pitch increase and (d) pitch decrease; and (e) pause cues (intervals of silence between statistical words). We compared these five individual prosodic changes to a baseline condition that included only statistical cues, i.e., transition probabilities between syllables (Figure 1). This comparison of multiple word segmentation cues, including contrasting prosodic cues, within a single study sets our study apart from previous speech segmentation studies.

We chose these five cues because pauses, durational, and pitch cues can function either as language-universal cues to word boundaries or as language-specific cues to word stress. Some of the cues have been shown to signal boundaries and word stress more successfully than others. Lengthening and pitch increase have been previously investigated in similar contexts (e.g., Saffran et al., 1996b; Frost et al., 2017; Ordin et al., 2017), but rarely in direct comparison (as in Tyler and Cutler, 2009). Most likely, past studies have focused on lengthening and pitch increase because both of these cues are typical acoustic correlates for expressing language-specific word-stress (Tyler and Cutler, 2009), and final lengthening is a cross-linguistic signal for word, phrase and sentence boundaries (Fletcher, 2010). Interestingly, shortened duration and decreased pitch have been neglected in past research on word segmentation (but see research on pitch decrease in a phrasal context; Mueller et al., 2010), presumably because these changes normally do not signal word stress. Still, they may provide valuable comparisons to lengthening and pitch increase to see if prosodic patterns that are not typical word stress correlates, and may even contrast with typical word stress correlates in natural languages, can still facilitate speech segmentation in an experimental setting. Further, the manipulation of acoustic cues that are not typical stress correlates of target words may lead to insights about how these cues may influence speech segmentation when occurring in a more distal prosodic context in real-life speech processing (cf. Dilley and McAuley, 2008).

Besides durational and pitch cues, intensity is a typical acoustic correlate of stress. We did not include intensity in our study because its role as a perceptual correlate of stress is unclear and because intensity levels are usually correlated with vowel quality and duration (Cutler, 2005; Ordin and Nespor, 2013).

Pauses, our third cue category, represent a language-universal boundary cue that should be salient independent of listeners' preferred stress patterns since they do not serve to signal word stress (Fletcher, 2010; Johnson, 2016). Pauses thus serve as reference cues for segmentation (Peña et al., 2002). Also, pauses are interesting because speech input consisting of words separated by pauses may help infant word learning less than continuous speech (Johnson et al., 2013). Crucially, pauses have a durational component and can be longer or shorter, but we regard them as a separate cue category because they differ from our syllable durational cues (lengthening and shortening) in many other aspects. For example, silent pauses do not consist of any acoustic material and thus cannot signal word stress.

We chose to focus on modifications of word-*final* syllables because in natural languages, final elements are often particularly susceptible to modifications (Swingley, 2009), e.g., in phrase-final lengthening (Fletcher, 2010), reduction of word-final unstressed syllables (Kohler and Rodgers, 2001; O'Brien and Fagan, 2016), or utterance-final pitch lowering in declarative sentences (Cruttenden, 1986; Hirst and Di Cristo, 1998). Also, pitch changes and durational changes implemented *on* word-final syllables can easily be compared to pause cues between words (that is, *after* word-final syllables). Modifying word-final syllables is also interesting insofar as this contrasts nicely with the dominant word stress pattern of our participants' native language, German, which carries stress predominantly on medial syllables of trisyllabic words (see below; Féry, 1998). If participants interpret the modified word-final syllables in our experiment as being stressed and relate this to the typical word-stress patterns of German, they may interpret the modifications to occur word-medially. This is particularly plausible for typical stress correlates such as pitch increase and lengthening, and may lead to a potential conflict between statistical cues (i.e., transition probabilities between syllables in the experimental speech stream) and prosodic cues. Such an effect would help to evaluate the relative influence of language-specific stress patterns and language-universal boundary cues on speech segmentation (cf. Crowhurst, 2016). Although it would certainly be interesting to test stress cues in other positions as well (Saffran et al., 1996b; Tyler and Cutler, 2009; Ordin et al., 2017; cf. Frost et al., 2017), the large number of acoustic cues we manipulated did not allow us to also investigate word-initial and word-medial changes.

### Word Stress in German

We focused on German, a stress-based language (Pamies Bertrán, 1999) that suits itself to theoretically grounded predictions, but is relatively underrepresented in speech segmentation research. Fortunately, a few speech segmentation studies on German (e.g., Bhatara et al., 2013; Ordin and Nespor, 2016; Ordin et al., 2017; Marimon Tarter, 2019), were available to inform our predictions and stimulus choice. In German, word stress in trisyllabic words is variable and depends on syllable structure (for in-depth discussions, see e.g., Delattre, 1965; Giegerich, 1985; Féry, 1998; Dogil and Williams, 1999; Domahs et al., 2014). Still, crucially, about half of all German trisyllabic words are stressed on their medial syllable, and word-initial or word-final stress occur less frequently (Féry, 1998). Similar relations hold for the syllable structures used in our study (see methods section; Féry, 1998; Ernestus and Neijt, 2008; Domahs et al., 2014). Thus, to the extent that listeners are sensitive to statistical regularities in speech, they should assume word-medial stress as the default German stress pattern when encountering new lexical items. If the stress pattern of our listeners' native language affects cue perception, this predicts that stress cues implemented on medial syllables of trisyllabic words should be perceived as converging with statistical cues (transitional probabilities between syllables), whereas stress cues implemented on word-initial or word-final syllables should be less convergent and may even conflict with statistical cues. Thus, German stress patterns contrast nicely with proposed language-universal cues such as phrase-final or sentence-final lengthening (e.g., Fletcher, 2010). If native German speaking listeners attend to a language-universal final lengthening cue, rather than to their dominant native stress pattern, our listeners should perceive word-final lengthening as a cue that strongly converges with the statistical cues, i.e., the transitional probabilities in the speech stream.

In German speech, stressed syllables are both longer and higher pitched than unstressed syllables (Ordin et al., 2017), but evidence about which of these two manifestations plays a bigger role for production and perception is inconclusive (pitch: Isachenko and Schädlich, 1966; syllable duration: Dogil and Williams, 1999; Nespor et al., 2008; Féry et al., 2011; Kohler, 2012; El Zarka et al., 2017). There are previous indications that in German, lengthening cues are perceived as converging with statistical cues when they occur in word-final position (Ordin and Nespor, 2013, 2016; Ordin et al., 2017), possibly because the cross-linguistic tendency to lengthen word final syllables (e.g., Fletcher, 2010) overrides the perception of the typical German word-medial stress pattern in these cases. Thus, German speakers may focus on pitch as a more reliable cue to word stress instead (cf. Kohler, 2012 on perceptual correlates of stress in German; Nespor et al., 2008; Féry et al., 2011), though this has not been observed experimentally (Ordin and Nespor, 2016).

Finally, testing opposing changes, such as lengthening vs. shortening of duration, or increase vs. decrease of pitch, represents a potentially important extension to previous findings on word segmentation in German, where only one direction of change in these cues was tested, because results will show whether *any* arbitrary durational or pitch modification acts as a segmentation cue (e.g., due to difference of any sort), or whether the directionality of the changes is important. To our knowledge, neither opposing cues nor pause cues have previously been tested in word segmentation experiments with German adults. Thus, overall, both theoretical and empirical considerations make German a particularly interesting language for our study.

### Hypotheses and Predictions

Our experimental setup given our chosen acoustic parameters leads to several hypotheses and predictions. The first hypothesis is that native German speaking listeners will interpret prosodic cues that occur either on (for durational and pitch cues) or after (for pause cues) the final syllable of statistical words as boundary signals that support the statistical cues already available (cue convergence). This predicts that adding prosodic cues on the word-final syllables will improve listeners' speech segmentation compared to their performance based on statistical cues alone. We refer to this hypothesis, where statistical cues and the individual prosodic cues are perceived as converging, as the “cue convergence hypothesis.”

The cue convergence hypothesis can be put forward for each of our prosodic cues separately, though it is more plausible for some changes than for others. Pause cues might be associated with word boundaries because in everyday speech, perceptible pauses occur almost exclusively at word boundaries, and hardly ever within words (Trainor and Adams, 2000; Fletcher, 2010; Sohail and Johnson, 2016; Matzinger et al., 2020). Lengthened syllables might also serve as signals for word-finality because domain-final elements are lengthened in everyday speech language-universally (Oller, 1973; Klatt, 1975; Vaissière, 1983; Tyler and Cutler, 2009; Fletcher, 2010; but also: White et al., 2020). Although domain-final lengthening mostly happens at the sentence or phrase level, we predict that it will generalize to the word level in our study, because in our design each statistical word is essentially a phrase, and there is evidence for successful speech segmentation based on final lengthening cues from previous speech segmentation experiments in several languages, including German (e.g., Saffran et al., 1996b; Tyler and Cutler, 2009; Ordin and Nespor, 2016; Frost et al., 2017; Ordin et al., 2017).

**Figure 2:**
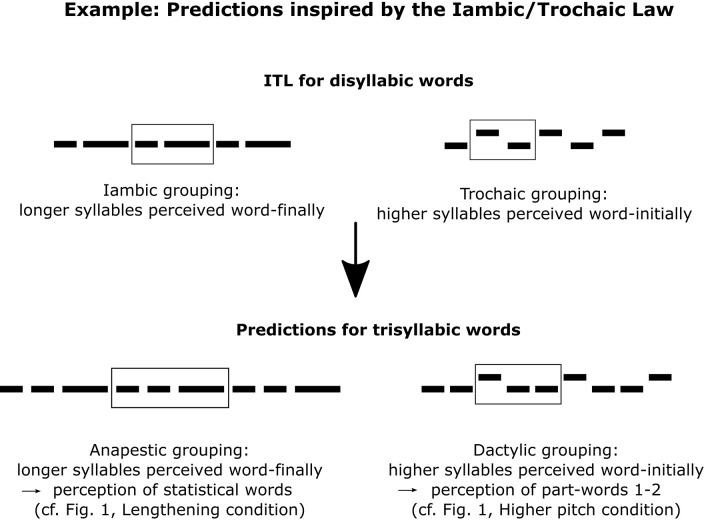
The Iambic/Trochaic Law (ITL) for disyllabic words leads to predictions for how listeners might perceive lengthened and/or higher-pitched syllables in trisyllabic words in our study. Horizontal black bars denote syllables.

Furthermore, the putatively language-independent Iambic/Trochaic Law (= ITL; Bolton, 1894; Woodrow, 1909; Hayes, 1995; Hay and Diehl, 2007; De la Mora et al., 2013; Frost et al., 2017; but see Iversen et al., 2008) states that listeners group sounds with longer duration as sequence-final (iambic grouping). Although the ITL focuses on disyllabic words, it can also be generalized to trisyllabic words (Trainor and Adams, 2000; Frost et al., 2017), supporting the prediction that final lengthening cues will converge with the available statistical cues and facilitate speech segmentation (Figure 2). In contrast, shortened syllables might also potentially signal word boundaries because, in natural languages, word-final elements are frequently phonetically reduced (Kohler and Rodgers, 2001; O'Brien and Fagan, 2016). This is because word processing is incremental and word-final elements are often highly predictable and thus not as informative for word identification as word-initial elements (Dahan and Magnuson, 2006; Swingley, 2009; Wedel et al., 2019).

Pitch decreases may signal word-finality because a sentence-final or phrase-final pitch decrease is very common in natural languages (Vaissière, 1983; Hirst and Di Cristo, 1998; Langus et al., 2012). Again, because in our study design each statistical word equals a phrase, this may generalize to the word level in our study. Finally, word-final pitch *increase* has also been shown to facilitate word segmentation in French, a language with word-final stress (Bagou et al., 2002; Tyler and Cutler, 2009), but not in German adults (Ordin and Nespor, 2016). Thus, overall, all five prosodic changes might potentially converge in word-final position with statistical cues, i.e., transition probabilities, and facilitate word segmentation. However, because of the perceptual salience of pauses, the abundant previous evidence for final lengthening (e.g., Ordin and Nespor, 2016; Ordin et al., 2017) and more tentative evidence against final pitch increase (Ordin and Nespor, 2016) as a speech segmentation cue in German, we predicted the cue convergence hypothesis to apply most strongly for pauses and lengthening, moderately strongly for pitch decrease, and less so for shortening and pitch increase.

An alternative to the cue convergence hypothesis is that native German speaking listeners may interpret prosodic cues implemented on the final syllable of a trisyllabic statistical word as conflicting with the statistical cues provided by the transition probabilities in the speech stream. If participants perceive the modified syllables as being stressed, and then group the syllables in the speech stream in a way that matches the predominant word-medial stress pattern of German (Norris and Cutler, 1988; Cutler, 1990; Cutler et al., 1992; Ordin et al., 2017), the prosodic modifications would then conflict with statistical cues. Since most German trisyllabic words are stressed on the medial syllable, this “cue conflict” hypothesis predicts that placing stress cues on the final syllable of the statistically defined words should bias German listeners' toward a different speech segmentation pattern than that based on statistical cues. Instead, they should group the modified syllables word-medially (see Figure 1, Parts 2b, 2d and 2e). We refer to this hypothesis as the “cue conflict hypothesis.”

The cue conflict hypothesis is plausible for typical correlates of stress, i.e., lengthening and pitch increase (Thiessen and Saffran, 2003; Johnson and Seidl, 2009), and less so for shortening and pitch decrease. Still, given abundant evidence from previous speech segmentation experiments in several languages, word-final lengthening is expected to converge with the statistical cues, overriding the tendency of native German speaking listeners to interpret lengthening as a cue to word stress (e.g., Ordin and Nespor, 2016; Ordin et al., 2017). Instead, native German speaking listeners are predicted to mostly use pitch increase as a cue for word stress, which would lead to a cue conflict with statistical cues for pitch increase only (contra Ordin and Nespor, 2016). Also, according to the ITL (Hayes, 1995; Nespor et al., 2008; Bion et al., 2011; De la Mora et al., 2013; Abboub et al., 2016), cross-linguistically, listeners group sounds with a higher pitch as sequence-initial (trochaic grouping). Thus, word-final pitch increase might conflict with statistical cues and lead to a different speech segmentation pattern (Figure 2). Furthermore, if listeners associate certain prosodic changes with word-final syllables (as per the cue convergence hypothesis), they should accordingly associate opposing changes with non-final syllables. Thus, if e.g., lengthening or pitch decrease on the final syllable of statistical words facilitate speech segmentation, the opposing changes (shortening or pitch increase, respectively) can be predicted to lead to a modified segmentation pattern.

In conclusion, for each prosodic cue, both hypotheses might reasonably be expected to hold, but overall, the preponderance of existing evidence suggests that pauses, final lengthening and final pitch decrease will lead to cue convergence, and final shortening and final pitch increase will conflict with statistical cues.

Regarding the relative effects of different prosodic cues, we hypothesized that pauses should have a bigger impact on word segmentation than other prosodic cues. Pauses may provide more salient signals than the other prosodic cues because they involve a highly perceptible decrease in signal amplitude (Fletcher, 2010; Friederici and Männel, 2013). Also, long enough pauses can make a word appear isolated. We thus predicted that word segmentation performance should show a greater increase with pause cues inserted between the “correct” statistical words than for our other prosodic changes. Beyond that basic prediction, durational cues and pitch cues might have different relative strengths, but we had no clear predictions about directionality, given weak and partly inconclusive previous data (cf. Tyler and Cutler, 2009), with some evidence for a durational preference (Männel and Friederici, 2016) and other evidence for a pitch preference (Ordin et al., 2017).

### Experimental Variations

Recently, many psychological findings have been found to be non-replicable, commonly known as the replication crisis (Shrout and Rodgers, 2018). Common reasons for a lack of replicability and generalizability are that experimental results are not robust to minor methodological changes (Munafò and Smith, 2018). To counteract this problem in our study, we conducted three experiments that examined whether participants would use similar segmentation strategies when testing paradigm and testing context varied. Our main aim was to evaluate the robustness of our results, and not to pin down effects of specific methodological differences. Therefore, our prime goal was not to design experiments that varied only in a single, carefully controlled methodological feature, but rather to have a spectrum of methods, in a single publication, that roughly mirror the methodological variation typifying previously published speech segmentation studies.

The three experiments implemented the same stimulus manipulations, but differed slightly in experimental setup. Experiment 1 was our initial pilot study, carried out in the participants' normal study or office environment; this study had minimal auditory memory requirements, and combined auditory and visual modalities, i.e., participants could see the test stimuli while they listened to the speech stream. This experiment addressed whether attested laboratory results replicate in an environment where background noise and visual distraction more closely resembled a real-life language learning context. Experiment 3 resembled existing speech segmentation experiments most closely (e.g., Tyler and Cutler, 2009; Frost et al., 2017; Ordin et al., 2017): it was done in a laboratory setting, exclusively in the auditory modality (similar to real-life first language acquisition), and thus involved a strong auditory memory component. However, in contrast to our experiment, where participants decided for single test stimuli if they were statistical words or part-words, most previous adult studies used a two-alternative forced choice testing procedure in which participants had to decide from a set of two test stimuli which of them was a word and which a part-word (see methods for Experiment 3 below). Experiment 2 was designed to be intermediate between Experiments 1 and 3. It was carried out in a laboratory setting, but involved auditory and visual modalities, with minimal memory components. We predicted that the effects of adding prosodic cues to the speech stream might unfold more strongly in experiments with syllables spoken by a native German speaker and a minimal memory component because the overall cognitive load is lower, and statistical cues are less prominent. Also, we expected all effects to be stronger in the laboratory, where people were less distracted than in a natural testing environment (cf. Toro et al., 2005; Erickson and Thiessen, 2015). Nonetheless, if the effects observed are robust and generalizable, they should occur—though perhaps less prominently—both in the natural environment in Experiment 1 because real language learning typically happens in a natural environment, and with an added memory component in Experiment 3 because language learning obviously involves memory (Palmer and Mattys, 2016; Wen, 2016; Pierce et al., 2017).

Additionally, syllables in Experiment 1 were recorded by a native speaker of English, whereas syllables in Experiments 2 and 3 were recorded by a native speaker of German. It is possible that sub-phonemic cues in the native English syllables may influence participants to rely less on their implicit knowledge of German prosody in Experiment 1 than in Experiments 2 and 3 (Quam and Creel, 2017). However, again, if the effects studied in our series of experiments are robust and generalizable, they should also occur in Experiment 1.

## General Methods

### Experimental Paradigm: Overview

We conducted three individual experiments with adult listeners. All three were artificial language learning experiments following an established experimental paradigm (e.g., Saffran et al., 1996a,b, 1999; Frost et al., 2017). Participants in all three experiments listened to a continuous speech stream that was created from four randomly generated trisyllabic pseudo-words making up an artificially constructed pseudo-language, and had to decide for each of 12 test stimuli whether they were “words” of the artificial pseudo-language or not. The study protocol was approved by the ethics board of the University of Vienna (reference number: #00333/00385), and all participants gave written informed consent in accordance with the Declaration of Helsinki.

### Experimental Conditions: Overview

**Figure 3:**
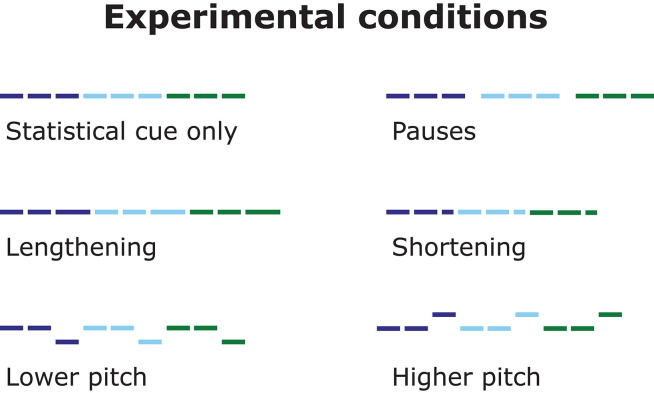
Overview of the experimental conditions. For each condition, the figure shows an example speech-stream of three words. Lines denote syllables, line length indicates duration, and line height pitch. Colors denote statistical words.

In the three experiments, we addressed the influence of different prosodic cues on word segmentation in a baseline and five prosodic conditions, resulting in six conditions in total (see Figure 3). In each prosodic condition, the speech stream was manipulated differently to check if that would provide cues to the segmentation of the words from the stream. These changes were always applied after (for pauses) or on (for duration and pitch) the final syllable of each trisyllabic word in the baseline statistical speech stream. Individual syllables were recordings of the same female speaker, but all manipulations of these basic syllables were precisely controlled by computer (for details see “Stimuli,” below).

**1. Statistical cue only condition (baseline condition)**. The only cue indicating word segmentation in the baseline condition was that syllable pairs within words had higher transitional probabilities than syllables crossing word-boundaries. Syllables within a word always co-occurred, resulting in within-word transitional probabilities of 1.0. In contrast, each word was pseudo-randomly followed by any of three different words, yielding a between-word syllable transitional probability of 0.33. Thus, participants could potentially infer that syllable pairs that occur more frequently together constitute a word, and those that co-occur less frequently do not. This statistical information was present in all conditions. Each syllable was normalized to a duration of 500 ms and a fundamental frequency of 210 Hz (for details see “Stimuli,” below). Typical syllable durations in speech stream experiments conducted in a laboratory are shorter than 500 ms (e.g., Saffran et al., 1996a; Tyler and Cutler, 2009; Frost et al., 2017; Ordin et al., 2017), but since we expected attentional capacities to be limited in Experiment 1, which was conducted in a natural environment, we chose a slow speech rate (2 syllables/second; Song et al., 2010; Palmer and Mattys, 2016) more typical for infant directed speech. This was expected to facilitate speech segmentation.

**2. Pause condition**. This condition was identical to the baseline condition, with the exception that in addition to the statistical cues, a short pause (250 ms) was inserted after each statistical word. We chose a pause duration of 250 ms because this duration is frequently chosen as a lower detection threshold in studies investigating the occurrence and perception of speech pauses (e.g., Zellner, 1994; Kahng, 2014).

**3. Lengthening condition**. This condition was identical to the baseline condition, except that in addition to the statistical cues, the final syllable of each word was lengthened by 50%, yielding a duration of 750 ms (cf. previous lengthening by ~40%: Saffran et al., 1996b; Ordin and Nespor, 2016; Frost et al., 2017; Ordin et al., 2017; or lengthening by 67%: Thiessen and Saffran, 2003). The duration of this additional lengthening was therefore identical to the pause duration in the pause condition.

**4. Shortening condition**. This condition was identical to the baseline condition, except that in addition to the statistical cues, the final syllable of each word was shortened by 50%, i.e., by the same proportion as syllables were lengthened in the lengthening condition, and thus had a duration of 250 ms.

**5. Higher pitch condition**. This condition was identical to the baseline condition, except that in addition to the statistical cues, the pitch of the final syllable of each word was increased to 260 Hz, making it 50 Hz higher than the pitch of all other syllables (cf. Thiessen and Saffran, 2003; Tyler and Cutler, 2009).

**6. Lower pitch condition**. This condition was identical to the baseline condition, except that in addition to the statistical cues, the pitch of the final syllable of each word was decreased to 160 Hz, making it 50 Hz lower than the pitch of all other syllables (as per the higher pitch condition).

## Experiment 1: Pilot Study

### Participants and Experimental Procedure

We tested 202 participants (19% male, mean age: 25.26), who were all native speakers of German and reported no auditory impairments. We used a between-subjects design: each participant was tested on one of six experimental conditions only (33 participants each in the pause, lengthening, and higher pitch condition; 34 participants each in the statistical cue only condition and the shortening condition; 35 participants in the lower pitch condition). Experimenters recruited the participants individually at the campus of the University of Vienna and they were tested *in situ* (e.g., in hallways, offices, public seating areas, etc.), while sitting or standing. Testing was performed with mobile testing equipment, i.e., a laptop computer and Sennheiser HD206 over-ear headphones. We ensured that the environment was free from obvious loud noise, but some background noise of other people walking by or chatting was unavoidable. We think that the effect of this background noise was minimal because participants could self-adjust the volume of the speech stream; none of them reported difficulties hearing the sounds.

Prior to the start of each experiment, participants were told that they would participate in an “Alien Language Learning Study” (as e.g., in Kirby et al., 2008), in which they would listen to a speech stream of an artificial pseudo-language and should decide for a set of 12 test stimuli whether they considered these to be “words” of the artificial language or not. Before listening to the speech stream, participants received a sheet of paper with all 12 test stimuli and were orally instructed in a standardized way to use a pen to circle the “words” of the “alien language” that they were about to hear. Participants listened to the speech stream for ~1 min (see “Stimuli” below for the precise lengths) and rated the 12 test stimuli simultaneously. Typical exposure lengths in speech stream experiments conducted with adults in a laboratory are slightly longer, but because we tested in a natural environment, where it may be hard to concentrate during longer exposure times, we chose a shorter exposure time more typical for infant experiments (Saffran et al., 1996a; Thiessen and Saffran, 2003; Erickson and Thiessen, 2015) and compensated for this difficulty by using a rather low speech rate (see above; Song et al., 2010; Palmer and Mattys, 2016). These parameters were expected to facilitate speech segmentation in a natural environment. Including instructions, the overall experimental procedure lasted for ~5 min. Immediately after participation, there was a short debriefing and participants' questions about the study were answered. Participants received no financial reward.

### Stimuli

The artificial pseudo-language consisted of four words with three CV (consonant-vowel) syllables each (Table 1, column 1, “Language 0”). The CV syllables were created from a pool of four vowels (a, u, i, o) and seven consonants (p, t, k, b, d, g, n). We ensured that the words created from this pool did not contain identical syllables, and were not existing words in German or English (which our participants spoke as a second language).

**Table 1:** Artificial words used for the different artificial pseudo-languages in the three experiments.

**Experiment 1**	**Experiments 2 & 3**			
**Language 0**	**Language 1**	**Language 2**	**Language 3**	**Language 4**
/batuki/	/bakupo/	/pifoke/	/dafego/	/mabopi/
/togabi/	/delaru/	/rovali/	/pebomi/	/veduka/
/punido/	/fumesi/	/nusema/	/kirune/	/sigale/
/dapiku/	/gonite/	/tabigu/	/lutiva/	/tonifu/

For the creation of the continuous speech streams of each condition, the four words were pseudo-randomly concatenated, with the restriction that no word could occur twice in a row. Each word was followed by each of the three remaining words equally often, which led to between-word transition probabilities of 0.33. One speech stream consisted of 40 words (i.e., each of the four words occurred 10 times in the stream). Depending on the condition, this led to total durations of the speech stream of 50 s (shortening condition), 60 s (baseline condition, lower pitch condition, and higher pitch condition), or 70 s (pause condition and lengthening condition).

The twelve test stimuli consisted of different *stimulus types*: four of the test stimuli were statistical *words*, i.e., the words that made up the particular artificial pseudo-language, and eight of the test stimuli were statistical *part-words*. Part-words could be of two different part-word classes and were created from syllables across word boundaries: either from the final syllable of a word and the initial and medial syllable of the following word (henceforth *part-words 1-2)*, or from the medial and final syllable of a word and the initial syllable of another word (henceforth *part-words 2-1*). Thus, crucially, in part-words 1–2, the original final syllables, which carried a prosodic cue in experimental conditions, occurred word-initially, and in part-words 2–1, the original final syllables occurred word-medially (see Figure 1). This procedure yielded 12 possible part-word stimuli in each part-word class (see Table 2 e.g., of part-words of language 1). As actual test stimuli, we selected four different stimuli of each part-word class, namely /ku-toga/, /ki-puni/, /do-toga/, and /bi-dapi/ as part-words 1–2, and /tuki-pu/, /piku-ba/, /tuki-da/ and /nido-ba/ as part-words 2–1.

**Table 2:**
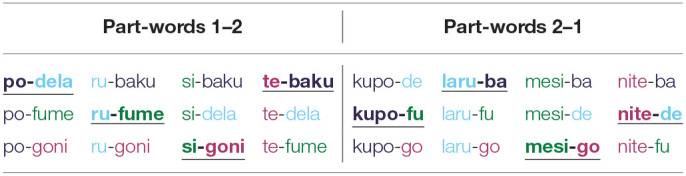
All possible part-words of pseudo-language 1, which consists of the words *bakupo, delaru, fumesi, gonite*.

*Part-words that share the same word-initial syllables are grouped in columns. An example of one possible selection of part-words for the test phase is underlined*.

To create the actual sound stimulus, each syllable was recorded by a female native speaker of American English. Each syllable was recorded individually in order to avoid co-articulation between syllables within a word (coarticulation could serve as an additional cue to speech segmentation, as e.g., in Johnson and Jusczyk, 2001, modifying the effects of the individual prosodic cues). The acoustic parameters of each syllable were modified using Praat (version 6.0.36; Boersma and Weenik, 2017), and the output syllables were then concatenated using custom code written in Python 3.6.3 to create the speech streams.

The acoustic modifications of the syllables concerned their fundamental frequency (“pitch”), duration, and amplitude. Pitch and duration of the syllables were modified using the pitch-synchronous overlap add (PSOLA) algorithm, which is a signal processing technique used for speech processing and synthesis implemented in Praat (Moulines and Charpentier, 1990). We used customized Praat scripts, which were based on the Praat functions “Manipulate → Replace Pitch Tier” and “Manipulate → Replace Duration Tier” to change syllable pitch and duration. For each syllable in the baseline condition, the fundamental frequency (*f*_0_) was normalized to a mean of 210 Hz, and the duration of each syllable was normalized to a mean of 500 ms. Durational changes were applied to the entire syllable except for the first 20 ms. This was done to avoid changes in voice onset time and associated consonant shifts. For the experimental conditions, all syllables were manipulated according to the same procedure to meet the respective duration and pitch specifications (see chapter 2.2). Syllable amplitude was made consistent by scaling the amplitude of each syllable so that its absolute peak amplitude was 0.99 (in Praat: Sound → Modify → Scale peak → New absolute peak: 0.99).

To avoid possible cueing to word boundaries, the continuous speech streams had a gradual fade-in and fade-out over the first and last five words, respectively, so that the perceived start and the end of the speech stream did not align with word boundaries. For the fade-in, the amplitude of the first 15 syllables, i.e., of each syllable of the first five words of the stream, was increased by 6.66% of the peak amplitude, so that at the beginning of the sixth word, the full amplitude was reached. Similarly, for the fade-out, we decreased the amplitude of each of the last 15 syllables by 6.66% of the peak amplitude. Amplitude manipulation was implemented in Python and Praat.

### D' Analysis and Results

**Figure 4:**
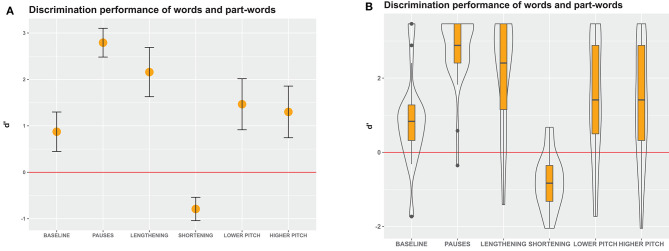
D' measures in Experiment 1, an immediate-decision task where participants decided whether visually presented letter strings were words that they had perceived in the speech stream or not. **(A)** Mean and 95% confidence intervals of participants' responses. Non-overlapping confidence intervals indicate significant differences between the groups. Confidence intervals that do not include 0 indicate significant differences from chance performance. **(B)** Boxes depict medians and quartiles, whiskers minimum and maximum values, and black dots outliers. Violin shapes around the boxes depict the distribution of d' values. The width of the violin shapes at a given y coordinate corresponds to the number of d' values in this region. Red lines: chance level performance.

To obtain a general overview of the influence of experimental conditions on participants' discrimination of words and non-words, we used signal detection theory measures and calculated d' values (Green and Swets, 1966; Macmillan and Kaplan, 1985; Macmillan and Creelman, 2005), based on hit rates (i.e., selection of statistical words as words) and false alarm rates (i.e., selection of statistical part-words as words). Perfect performance (100% hits and 0% false alarms) causes mathematical problems in signal detection theory, requiring *post-hoc* changes to these values to avoid divide-by-zero issues when calculating d prime values. Therefore, we adjusted perfect hit rates and false alarm rates according to the standard 1/(2N) rule, which adds 1/(2N) to proportions of 0 and subtracts 1/(2N) from proportions of 1 (Hautus, 1995; Stanislaw and Todorov, 1999; Brown and White, 2005; Macmillan and Creelman, 2005). D' values of 0 indicate that participants selected words and non-words at chance level, d' values above 0 indicate a discrimination performance above chance (i.e., participants perceived many statistical words as words), and d' values below 0 indicate a discrimination performance worse than chance (i.e., participants perceived many statistical part-words as words). We computed 95% confidence intervals (CIs; Figure 4) to determine if the differences between individual groups and the differences to chance level performance were significant. Confidence intervals that do not overlap with each other indicate significant differences between groups. Confidence intervals that do not include d' values of 0 indicate that word perception is either better (CIs above 0) or worse (CIs below 0) than chance (Cumming and Finch, 2005; Cumming, 2012, 2014).

Our calculation of d' values revealed that in the baseline condition, participants performed better than chance (Figure 4), indicating that statistical cues alone sufficed to detect words in the speech stream. In the pause and lengthening conditions, participants excelled on the task, indicating that pauses and final lengthening provided strong convergent cues for speech segmentation. In the pause and lengthening conditions, the participants' performance was also significantly higher than in the baseline condition, indicating that adding these cues to a speech stream significantly facilitates segmentation performance.

In contrast, in the lower and higher pitch conditions, participants showed only moderate discrimination performance, which was above chance but did not significantly differ from the baseline statistical condition. This suggests that enhancing statistical cues with a pitch modification on the word-final syllable did not appreciably aid speech segmentation for our listeners. Interestingly, in the shortening condition, the performance was in fact very poor and significantly worse than baseline, showing that shortening final syllables hindered word segmentation. This suggests that prosodic cues can override statistical cues when they conflict, but does not yet show if the low performance was due to participants perceiving the shortened syllable in word-initial (part-words 1–2) or word-medial (part-words 2–1) position. To clarify this, we conducted a more fine-grained analysis involving a generalized linear mixed model.

### Generalized Linear Mixed Model: Analysis

To investigate if the different prosodic cues had an effect on which stimulus type (statistical word or one of the two statistical part-word types) the participants perceived, i.e., on the “correctness” of their responses on the three different stimulus types, we fitted a logistic Generalized Linear Mixed Model (Baayen, 2008) with logit link function (McCullagh and Nelder, 1989). *Condition* and *stimulus type*, as well as their interaction, were included as fixed effects into the model. We also entered a random intercepts effect of *participant* in the model. To avoid inflated type I error rates we included a random slope (Schielzeth and Forstmeier, 2009; Barr et al., 2013) of *stimulus type* within *participant*. Before including this factor into the random slope we manually dummy coded and then centered it. The sample size for this model was 2,424 data points (202 individuals tested on one condition each, with 12 trials), 1,719 of which were correct responses. Responses were coded as “correct” when participants selected the statistical words as being “words” and rejected the statistical part-words as being “words” of the artificial language, so that for each stimulus type, perfect performance would be 100%, and chance-level performance (guessing) would be 50% correct responses.

The model was fitted in R (version 3.6.0; R Development Core Team, 2018), using the function *glmer* of the R-package *lme4* (version 1.1.21; Bates et al., 2015) and the optimizer “bobyqa”.

To test the overall significance of *condition* (i.e., its main effect and its potential interaction with *stimulus type*), we used a likelihood ratio test to compare our full model to a null model that was identical to the respective full model except for that it did not include *condition* and its interaction with *stimulus type* (R function *anova* with argument “test” set to “Chisq”; Dobson, 2002).

*P*-values for the effect of individual predictors are based on likelihood ratio tests that compare the full model with respective reduced models lacking the effects one at a time (R function *drop1*; Barr et al., 2013). We determined model stability by dropping individuals one at a time and comparing the estimates obtained for these subsets with those obtained for the full data set, which revealed that our model was fairly stable (see Supplementary Table 1). We determined confidence intervals of estimates and the fitted model using a parametric bootstrap (function *bootMer* of the package *lme4*, using 1,000 parametric bootstraps).

### Generalized Linear Mixed Model: Results

**Figure 5:**
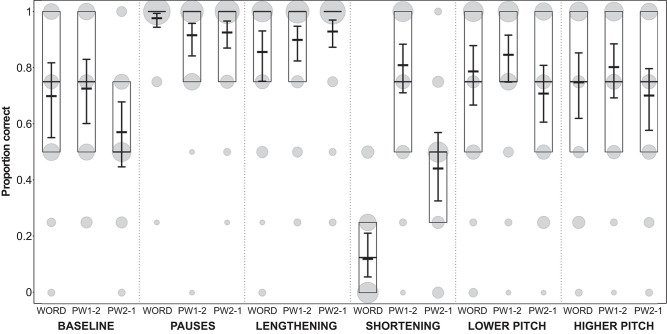
Proportion of participants' correct answers in Experiment 1. Proportions are displayed for each condition and each stimulus type (WORD, word with modified syllable in final position; PW1-2, part-word with modified syllable in initial position; PW2-1, part-word with modified syllable in medial position). Model results: thick horizontal black lines, with error bars depicting the bootstrapped 95% confidence intervals. Boxes depict medians and quartiles, and gray dots the actual observations (the area of the dots indicates the number of responses per combination of condition, stimulus type, and proportion correct).

Overall, the full model (for details, see Supplementary Table 1) was significantly different from the null model, indicating an effect of *condition* or its potential interaction with *stimulus type* on the perception of words in a speech stream (likelihood ratio test: χ^2^ = 147.865, df = 15, *p* < 0.001). Word perception was measured by the proportion of “correct” answers in the experiment, specifically, the proportion of statistical words and part-words that listeners identified as words and part-words, respectively. More specifically, we found that the interaction between *condition* and *stimulus type* had a significant effect on word perception (likelihood ratio test: χ^2^ = 63.129, df = 10, *p* < 0.001), indicating that the pattern of correct responses to words vs. part-words varied between conditions (see Figure 5). The computed confidence intervals (Figure 5) allow us to make comparisons between individual groups. This confirms the main results from the d' analysis above, and additionally allows comparisons between participant performance on the three different stimulus types.

In all conditions except the shortening condition, the performance on words, part-words 1–2 and part-words 2–1 was very similar (Figure 5), i.e., words were correctly selected as being statistical words and part-words were correctly rejected. Interestingly, in the shortening condition, our analysis (see model estimates and confidence intervals in Figure 5) revealed very clearly that performance on part-words 2–1 (i.e., stimuli where the shortened syllables occurred word-medially) was poor, because participants identified many part-words 2–1 as *words* (which count as false alarms in our analysis). That is, they interpreted the shortened syllables as being word-medial. This is a violation of typical German word stress because German word-stress usually occurs word-medially, and stressed syllables are usually lengthened (see Figure 1). Furthermore, performance on the statistically correct words (where the shortened syllables occurred word-finally) was also very poor, because participants incorrectly identified many of these words as *part-words*. However, they correctly identified most part-words 1–2 (i.e., stimuli where the shortened syllables occurred word-initially) as *part-words*. This clearly shows that in this condition participants were biased to perceive as “words” those stimuli where the duration of the medial syllable was shortened.

## Experiment 2

The main aim of this experiment was to replicate Experiment 1 in a more controlled laboratory setting. For the sake of this comparison, we kept the key aspects of Experiment 1, most notably that participants evaluated the test items on a sheet of paper while listening to the speech stream, but Experiment 2 was a within-subjects study that controlled more aspects of the experimental procedure via randomization than Experiment 1. Experiment 2 specifically focused on the conditions that significantly differed from the baseline in Experiment 1, namely conditions 1 to 4, and omitted the pitch manipulation.

### Participants and Experimental Procedure

We tested 34 participants (21% male, mean age: 24.85), who were all native speakers of German and reported no auditory impairments. Participants were recruited via posters or online advertisements. Participant instructions and the overall testing procedure were identical to Experiment 1, except that participants were now individually tested in a quiet laboratory setting. While sitting ~60 cm from a 13″ monitor, they were shown instructions and listened to the speech stream via an experimental interface created in PsychoPy (version 1.90.3; Peirce, 2007). Further, we used a within-subjects design, in which all participants were tested on all four conditions in a randomized order. The speech stream of each condition now lasted twice as long, for ~2 min (see “Stimuli” below for details). Between each condition, participants were given a 30 s break. No feedback on the responses was provided. Thus, including instructions and a final debriefing, the experiment lasted ~20–25 min. Participants were given modest monetary compensation for their participation.

### Stimuli

Because each participant was tested on four different experimental conditions, we created four different artificial pseudo-languages (Table 1, columns 2–5), consisting of four words with three CV (consonant-vowel) syllables each. For each participant, we pseudo-randomized which pseudo-language was used for which condition. We carefully controlled stimulus creation to avoid potential transfer or priming from words learned in one condition in one pseudo-language to words in another condition in another pseudo-language. Therefore, the CV syllables were created from a pool of five vowels (a, e, i, o, u) and 13 consonants, namely six stops (b, d, g, p, t, k), three fricatives (f, v, s), and four sonorants (m, n, l, r). In total, the four words of each language required 12 vowels and 12 consonants. To minimize possible cues resulting from the distribution of vowels and consonants we ensured that within each pseudo-language used in Experiments 2 and 3, vowels were evenly distributed (two of the vowels occurred three times and three of the vowels occurred twice) and that no word contained the same vowel twice. Also, no consonant occurred within one pseudo-language more than once. Thus, each syllable was unique within a pseudo-language. Moreover, across all four pseudo-languages, none of the syllables occurred more than twice, with the majority of the syllables only occurring once.

One speech stream consisted of 96 words (i.e., each of the four words occurred 24 times in the stream). Depending on the condition, this led to total durations of the speech stream of 120 s (shortening condition), 144 s (baseline condition), or 168 s (pause condition and lengthening condition).

As in Experiment 1, each participant received 12 test stimuli per condition, which consisted of statistical *words* and statistical *part-words*, created as described above for Experiment 1. For each participant and in each condition, the set of test stimuli included four statistical *words*. The four *part-words 1–2* and the four *part-words 2–1* were pseudo-randomly selected for each individual participant and each condition. We ensured that each first and second part was represented once in each part-word class (e.g., see words highlighted in bold in Table 2).

The actual sound signals of the speech streams were created as in Experiment 1, except that in this experiment the syllables from which the speech streams were created were recorded by a different female native speaker (in this case of German).

### D' Analysis and Results

**Figure 6:**
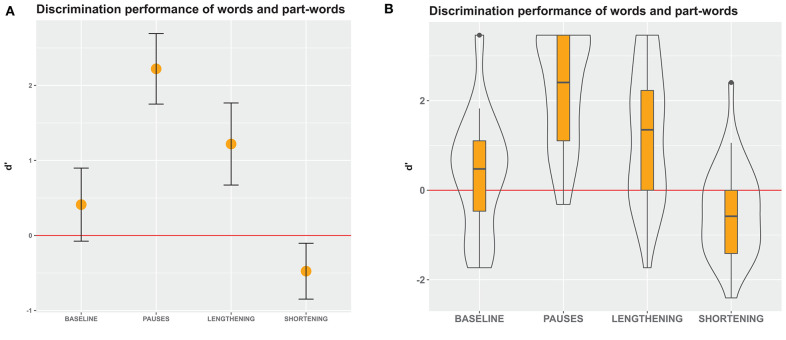
D' measures in Experiment 2, an immediate-decision task where participants decided whether visually presented letter strings were words that they had perceived in the speech stream or not. **(A)** Mean and 95% confidence intervals of participants' responses. Non-overlapping confidence intervals indicate significant differences between the groups. Confidence intervals that do not include 0 indicate significant differences from chance performance. **(B)** Boxes depict medians and quartiles, whiskers minimum and maximum values, and black dots outliers. Violin shapes around the boxes depict the distribution of d' values. The width of the violin shapes at a given y coordinate corresponds to the number of datapoints in this region. Red lines: chance level performance.

Our calculation of d' values (for details about the analysis, see Experiment 1) revealed that discrimination performance was best in the pause condition, moderately good in the lengthening condition and almost above chance in the baseline condition. Shortening again hindered speech segmentation compared to the baseline (Figure 6). Thus, the effects were similar to those in Experiment 1, but performance was worse. As in Experiment 1, we performed a generalized linear mixed model to investigate the reasons for the low performance in the shortening condition.

### Generalized Linear Mixed Model: Analysis

As in Experiment 1, we fitted a logistic Generalized Linear Mixed Model (Baayen, 2008) with logit link function (McCullagh and Nelder, 1989) to test whether the perception of words in the speech stream was influenced by condition and stimulus type (statistical word or one of the two statistical part-word types). We again included *condition* and *stimulus type*, as well as their interaction as fixed effects into the model. To control for the effects of *pseudo-language* (factor with four levels; participants were exposed to a different pseudo-language in each of the four conditions) and *order of the conditions* (covariate with values 0–3), we included them as further fixed effects. We also entered a random intercepts effect of *participant* in the model. Again, to keep type I error rates at the nominal level of 0.05, we included random slopes (Schielzeth and Forstmeier, 2009; Barr et al., 2013) of *condition, stimulus type*, their interaction, *order of the conditions*, and *language* within *participant*. Before including factors into the random slopes we manually dummy coded and then centered them. We did not include the correlations between random intercept and random slopes terms in the final model because an initial model including these correlations and thus being maximal with regard to random effects failed to converge. The control predictor *order of the conditions* was z-transformed (to a mean of zero and a standard deviation of one). The sample for this model was 1,632 data points (34 individuals tested on four conditions with 12 trials each), 1,066 of which were correct responses.

Significances of the individual predictors, model stability (for details see Supplementary Table 2) and confidence intervals were calculated as described for Experiment 1.

### Generalized Linear Mixed Model: Results

**Figure 7:**
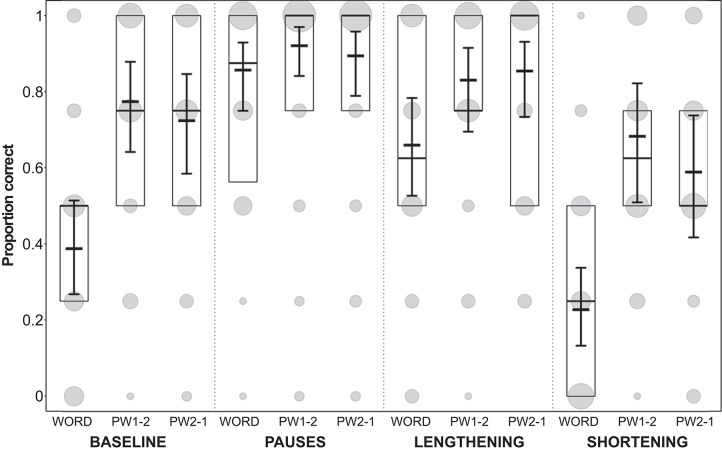
Proportion of participants' correct answers in Experiment 2. Proportions are displayed for each condition and each stimulus type (WORD, word with modified syllable in final position; PW1-2, part-word with modified syllable in initial position; PW2-1, part-word with modified syllable in medial position). Model results: thick horizontal black lines, with error bars depicting the bootstrapped 95% confidence intervals. Boxes depict medians and quartiles, and gray dots the actual observations (the area of the dots indicates the number of responses per combination of condition, stimulus type, and proportion correct).

In experiment 2, a comparison of the full model with the null model again revealed an effect of either *condition* or its potential interaction with *stimulus type* on the perception of words in a speech stream (likelihood ratio test comparing the full and the null model: χ^2^ = 63.00, df = 9, *p* < 0.001; for model details, see Supplementary Table 2). Exploring these effects, we found that the interaction effect between *condition* and *stimulus type* was non-significant (likelihood ratio test: χ^2^ = 11.329, df = 6, *p* = 0.079). However, because this interaction effect was very close to being significant, it is not justified to exclude it from the model and determine the effect of *condition* alone. Overall, these results again reflect different response patterns between conditions (see Figures 6, 7), but the differences between the conditions were not as prominent as in Experiment 1. Again, this confirms the main results from the d' analysis above. Although the interaction effect did not meet the threshold for statistical significance, comparisons between the three different stimulus types can shed light on the speech segmentation strategies employed in the different conditions and provide valuable comparison to experiments 1 and 3. With regard to the outcomes of experiment 1, we were most interested in the shortening condition, for which we predicted a low performance on words and part-words 2–1, and a high performance on part-words 1–2.

The comparison between the performances on the three different stimulus types (see model estimates and confidence intervals in Figure 7) revealed that in the pause condition, participants showed high performance on all stimuli (correctly identifying statistical words as *words*, and statistical part-words as *part-words*). In the baseline and the lengthening condition, participants performed rather well at identifying part-words, but relatively poorly identifying words. This indicates a bias to select only a few stimuli as words, leading to a considerable number of misses for words. In the shortening condition, participants again performed worst, missing many words, and labeling them as part-words incorrectly (see model estimates and confidence intervals in Figure 7), indicating cue conflict for this condition. The performance on part-words 1–2 and part-words 2–1 was similar, which indicates that participants perceived the shortened cue on the word-medial and word-initial syllable equally often.

The control predictors *order of the conditions* (likelihood ratio test: χ^2^ = 0.945, df = 1, *p* = 0.329) and *pseudo-language* (likelihood ratio test: χ^2^ = 1.725, df = 3, *p* = 0.631) did not have a significant effect on discrimination performance of words and part-words. The null effect of the predictor *order of the conditions* indicates that there was no cross-condition interference of segmentation strategies and participants did not infer a consistent rule that they transferred from condition to condition.

## Experiment 3

Given the overall consistent results of Experiments 1 and 2, the main goal of Experiment 3 was to probe their robustness, by modifying the paradigm. In particular, we added a more pronounced auditory memory component by delaying responses and presenting the test stimuli acoustically instead of visually. Participants first listened to the entire speech stream. Then, in a subsequent test phase, they listened to single probe stimuli and made a decision for each stimulus whether it was a word or a part-word. Correct responses thus required participants to remember any words that they perceived during presentation, despite interference from repeatedly hearing part-words during testing. Thus, Experiment 3 tested not just the effect of our manipulations on the immediate perception of test stimuli, but also how well people remembered them. This makes this experiment resemble real-life language learning more closely, and resembles many previous speech segmentation experiments (e.g., Tyler and Cutler, 2009; Frost et al., 2017; Ordin et al., 2017). In Experiment 3, we investigated all six experimental conditions from Experiment 1.

### Participants and Experimental Procedure

We tested 42 participants (26% male, mean age: 24.19 years), who were all native speakers of German and reported no auditory impairments. Participants were recruited via posters or online advertisements. As in Experiment 2, testing happened in a laboratory; the experiment was administered via an experimental interface created in PsychoPy (version 1.90.3; Peirce, 2007), which coordinated the presentation of instructions, speech streams and acoustic test stimuli, and collected key-press responses. We used a within-subjects design, in which all participants were tested on four of the six experimental conditions, namely the baseline condition, the pause condition, one of the durational cue conditions (either the lengthening or the shortening condition) and one of the pitch cue conditions (either the lower or higher pitch condition). Which of the durational and pitch cue conditions a participant ran was pseudo-randomized. We did not test participants on all six conditions to reduce the chance that they inferred a rule (e.g., “the modified syllable is always the last syllable of the word”) that might transfer from condition to condition. The presentation order of the conditions was randomized. Immediately after listening to each speech stream, participants listened to the corresponding 12 test stimuli in a randomized order and indicated, after each stimulus, whether they considered it to be a word in the preceding artificial language or not. Participants pressed a green-labeled key on a computer keyboard to indicate “word” and a red key if not. One half of the participants pressed the green key with the left hand and the red key with the right hand. To avoid effects of handedness, for the other half of the participants, this was reversed. No feedback on the responses was provided.

As in Experiment 2, the speech stream for each condition lasted for ~2 min (see “Stimuli” below for details), participants completed each test phase at their own pace, and between the conditions, participants were given a 30 s break. Thus, including instructions and a final debriefing, the experiment lasted ~20–25 min. Participants were given modest monetary compensation for their participation in the experiment.

### Stimuli

For Experiment 3, we used the same artificial languages (Table 1, columns 2–5), the same speech streams (including two additional speech streams for the two pitch conditions) and the same test stimuli as for Experiment 2 (e.g., see words highlighted in bold in Table 2). The acoustic versions of the test stimuli were created from syllables spoken by the same female native speaker of German as Experiment 2, in the same way as previous speech streams (see “Stimuli” in Experiment 1 and 2). All syllables were normalized to the default length of 500 ms and the default pitch of 210 Hz. The test stimuli did not carry any modifications of duration or pitch from these standards.

### D' Analysis and Results

**Figure 8:**
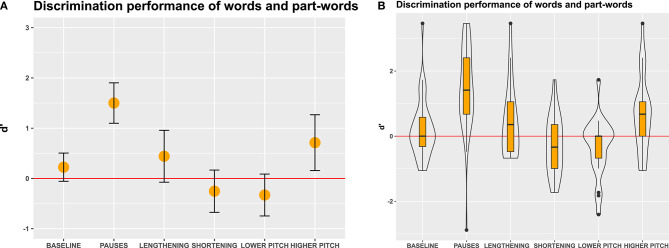
D' measures in Experiment 3, a decision task where participants decided whether acoustically presented stimuli were words that they had perceived in the speech stream or not. **(A)** Mean and 95% confidence intervals of participants' responses. Non-overlapping confidence intervals indicate significant differences between the groups. Confidence intervals that do not include 0 indicate significant differences from chance performance. **(B)** Boxes depict medians and quartiles, whiskers minimum and maximum values, and black dots outliers. Violin shapes around the boxes depict the distribution of d' values. The width of the violin shapes at a given y coordinate corresponds to the number of d' values in this region. Red lines: chance level performance.

Our calculation of d' values (for details about the analysis, see Experiment 1) revealed that pauses again significantly improved discrimination performance compared to the baseline (Figure 8). In all other conditions, discrimination performance was near chance level, except for the higher pitch condition, where it was slightly above chance. There was a tendency that participants discriminated words and part-words better than chance in the baseline and lengthening conditions and worse than chance in the shortening and lower pitch conditions. Thus, the directions of the effects were similar to Experiments 1 and 2, but all effects besides those of pauses were very weak. As in Experiments 1 and 2, we performed a generalized linear mixed model to investigate the reasons for the generally low performance.

### Generalized Linear Mixed Model: Analysis

As for Experiments 1 and 2, we fitted a logistic Generalized Linear Mixed Model (Baayen, 2008) with logit link function (McCullagh and Nelder, 1989) to test whether the perception of words in the speech stream was influenced by condition and stimulus type (statistical word or one of the two statistical part-word types). *Condition* and *stimulus type*, as well as their interaction were included as fixed effects into the model. To control for the effects of *pseudo-language* (factor with four levels; participants were exposed to a different pseudo-language in each of the four conditions), *order of the conditions* (covariate with values 0–3), and *trial number* (counting from 0 to 11 within each condition), these were included as additional fixed effects. The predictor *pseudo-language* was manually dummy coded with Language 1 being the reference category, and then centered. As in previous experiments, we entered a random intercept of *participant* in the model, and included random slopes (Schielzeth and Forstmeier, 2009; Barr et al., 2013) of *condition, stimulus type*, their interaction, *order of the conditions* and *trial number* within *participant*. Again, before including factors into the random slopes we manually dummy coded and then centered them. The correlations between random intercept and random slopes terms were not included in the final model because an initial model including these correlations—and thus being maximal with regard to random effects—did not converge. The control predictors *order of the conditions* and *trial number* were z-transformed (to a mean of zero and a standard deviation of one). The sample for this model consisted of 42 individuals tested on 4 conditions with 12 trials each. This yielded 2016 data points in total, 1,063 of which revealed a correct response.

Significances of the individual predictors, model stability (for details see Supplementary Table 3) and confidence intervals were calculated as described for Experiment 1.

### Generalized Linear Mixed Model: Results

**Figure 9:**
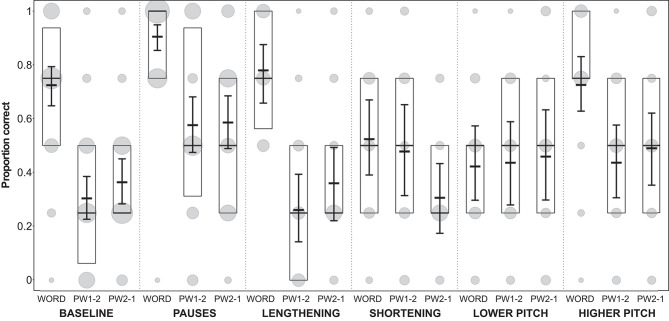
Proportion of participants' correct answers in Experiment 3. Proportions are displayed for each condition and each stimulus type (WORD, word with modified syllable in final position; PW1-2, part-word with modified syllable in initial position; PW2-1, part-word with modified syllable in medial position). Model results: thick horizontal black lines, with error bars depicting the bootstrapped 95% confidence intervals. Boxes depict medians and quartiles, and gray dots the actual observations (the area of the dots indicates the number of responses per combination of condition, stimulus type, and proportion correct).

As for Experiments 1 and 2, the comparison of the full model (for details, see Supplementary Table 3) and the null model for Experiment 3 revealed that *condition* or its potential interaction with *stimulus type* had an impact on the perception of words (likelihood ratio test: χ^2^ = 62.20, df = 15, *p* < 0.001). Unpacking these effects, we found that the interaction between *condition* and *stimulus type* had a significant effect on word perception (likelihood ratio test: χ^2^ = 31.963, df = 10, *p* < 0.001). This means that the pattern of correct responses on words and part-words varied between conditions (see Figures 8, 9). However, the overall results of Experiment 3 were slightly less clear than for Experiments 1 and 2. This confirms the main results from the d' analysis above.

Participants showed quite high performance on words and both part-word types in the pause condition, but they showed a high performance on words and a low performance on part-words in the baseline, lengthening and high pitch conditions. The rather low performance on part-words in these conditions indicates that participants had the tendency to select many incorrect stimuli as words, resulting in many false alarms for part-words. In the shortening and low pitch conditions, performance was rather low on words and part-words. Interestingly, as in Experiment 1, in the shortening condition, performance on part-words 2–1 was low, which indicates that participants had the tendency to perceive stimuli where shortening happened on the medial syllable as words (see model estimates and confidence intervals in Figure 9).

The control predictors *pseudo-language* (likelihood ratio test: χ^2^ = 4.013, df = 3, *p* = 0.260), *order of the conditions* (likelihood ratio test: χ^2^ = 0.159, df = 1, *p* = 0.692), and *trial number* (likelihood ratio test: χ^2^ = 0.014, df = 1, *p* = 0.907) did not have a significant effect on discrimination performance of words and part-words. The null effect of the predictor *order of the conditions* indicates that there was no cross-condition interference of segmentation strategies and participants did not infer a consistent rule that they transferred from condition to condition.

## Discussion

Our study indicates that manipulating prosodic information has clear effects on speech segmentation by adult German-speaking listeners, mostly improving performance relative to a statistics-only baseline (see non-overlapping confidence intervals in Figures 4, 6, 8). This basic result is consistent with considerable previously published data. A significant interaction between *condition* and *stimulus type* in Experiments 1 and 3 (and near significant interaction with *p* = 0.079 in Experiment 2) clearly shows that listeners' identifications of words and part-words differed in the different prosodic modification conditions. Our prosodic modifications occurred either on the final syllable of a trisyllabic nonsense word (for durational and pitch cues), or after it (for pauses). Our results further show that listeners interpreted different prosodic modifications as occurring at different positions in these trisyllabic words. This provides clear evidence that different prosodic cues have differing effects on speech segmentation, in an experiment where for the first time multiple prosodic cues were contrastively manipulated with other acoustic factors being closely controlled.

### The Positive Effects of Pauses and Final Lengthening on Speech Segmentation

Overall, adding pauses and lengthening the final syllable converged with the statistical cues, significantly facilitating speech segmentation based on statistical cues alone. In Experiment 1, participants identified most of the test items correctly in the pause and lengthening condition, whereas in the baseline condition with statistical cues alone, performance was only slightly above chance (Figure 4). In Experiment 2, pause and lengthening cues improved identification of words, but not the rejection of part-words, compared to the baseline condition (see non-overlapping CIs in Figure 7). In Experiment 3, which added a pronounced memory component, pauses, but not final lengthening, led to a higher performance compared to the statistical-cues-only condition (Figure 8). This overall convergent effect of final lengthening is consistent with the language-universal occurrence of domain-final lengthening, but not a language-specific stress pattern because German trisyllabic words typically do not carry stress on their final syllables (for a discussion, see Crowhurst, 2016). Overall, these results are in accordance with a large body of previous research showing that final lengthening cues are perceived as converging with statistical cues, thus facilitating speech segmentation (Saffran et al., 1996b; Tyler and Cutler, 2009; Ordin and Nespor, 2016; Frost et al., 2017; Ordin et al., 2017). These results are thus consistent with the cue convergence hypothesis for pause and final lengthening cues.

### The Negative Effect of Final Shortening on Speech Segmentation

In contrast, shortening the final syllable actively hindered the identification of statistical words, compared to statistical cues alone, consistent with the cue conflict hypothesis for final shortening cues. This was illustrated most clearly in Experiments 1 and 2, where identification of statistical words in the shortening condition was significantly lower than in the baseline condition (see Figures 4, 6). In Experiment 3, overall performance in the shortening condition was quite low because either “correct” statistical words were missed, or part-words were mistakenly selected as “words” (see Figures 8, 9). Interestingly, in Experiments 1 and 3, participants selected many part-words 2–1 as words (see low performance on part-words 2–1 in Experiments 1 and 3; Figures 5, 9), indicating that participants tended to perceive shortened syllables as occurring word-medially. Thus, when prosodic and statistical cues conflict, prosodic cues overrode statistical cues in the speech segmentation process, yielding “word” percepts based on prosodic patterns that conflict with those based on transition probabilities. Prosody has also overpowered statistics in previous studies: English infants grouped syllables with a combination of longer duration, higher pitch and higher intensity as word-initial, disregarding statistical cues (e.g., Johnson and Jusczyk, 2001; Thiessen and Saffran, 2003; Johnson and Seidl, 2009). In our study, however, neither final lengthening nor pitch increase overrode statistical cues when occurring individually (although final lengthening significantly augmented such cues), but shortening alone sufficed to override the statistical cues.

Final shortening may strongly influence speech segmentation because listeners have a language-universal preference for final lengthening (Tyler and Cutler, 2009; Fletcher, 2010; but also: Ordin et al., 2017; White et al., 2020). Encountering the opposite cue—final shortening—might thus actively interfere with word segmentation. The contrasting results we observed for final lengthening and shortening cues were consistent with our hypothesis that contrasting cues should have contrasting effects. Participants may also have perceived the shortening cues on the medial syllables because, when medial syllables are short, final syllables are perceived as longer, which would again fit the language-universal preference for final lengthening. Another potential explanation for the word-medial perception of shortened syllables might be that some German trisyllabic words do carry stress on the initial or final syllable (Domahs et al., 2014; Ordin and Nespor, 2016; Ordin et al., 2017) and that in these words, medial syllables may appear weaker and shortened. However, overall, it seems unlikely that language-specific word stress patterns explain why shortening was perceived on medial syllables because German trisyllabic words are typically stressed on the medial syllable (Domahs et al., 2014; Ordin and Nespor, 2016; Ordin et al., 2017), and shortening is not typically associated with stress (Tyler and Cutler, 2009; Ordin and Nespor, 2013). Thus, regarding duration, language-universal factors may play a bigger role for speech segmentation than language-specific word stress patterns (for a discussion, see Crowhurst, 2016).

### The Relative Strengths of Different Prosodic Cues

Turning to the relative strengths of the different prosodic cues, our study allows a precise quantitative evaluation of the effect of pauses, duration, and pitch manipulations relative to a common statistical baseline. Overall, pauses between words provided the most helpful cues for speech segmentation. Especially in Experiment 3, which involved a strong memory component and was thus the most challenging, pauses outranked most other cues in effect (see non-overlapping CIs for words in all but one cue in Figure 9). This may be because pauses involve an immediate and very salient decrease in signal amplitude, relative to the other cues we tested (Fletcher, 2010). Additionally, pauses should provide nearly unambiguous signals for word boundaries because, in real speech, pauses almost exclusively occur at word boundaries and are not as flexibly distributed as changes in duration or pitch (Trainor and Adams, 2000; Fletcher, 2010; Matzinger et al., 2020).

Besides pauses, durational cues proved to be highly relevant cues for speech segmentation. In Experiment 1, final lengthening aided segmentation roughly as much as pauses (see Figure 4, and overlapping CIs in Figure 5), and final shortening was powerful enough to override statistical cues entirely.

In contrast, and perhaps surprisingly given previous results, pitch cues did not have very strong effects: in Experiment 3, performance when word-final pitch was increased was higher than when it was decreased, but performance based on modified pitch did not differ significantly from baseline performance in any of our experiments (see overlapping CIs in Figures 4, 8). Thus, neither final pitch increase nor pitch decrease greatly affected speech segmentation. This result is concordant with the null effects of final pitch increase in German, Italian, Spanish and English (Toro et al., 2009; Tyler and Cutler, 2009; Ordin and Nespor, 2016), but contrasts with the facilitating effect of final pitch increase in French (most likely due to language-specific stress patterns of French: Tyler and Cutler, 2009). This may be because the pitch cues were perceived as neither converging or conflicting with the statistical cues, or because any perceived cue conflict was not strong enough to override the ever-present statistical cues. Investigations with more languages (especially tonal languages) employing a wider range of pitch changes would help resolve the role of pitch in word segmentation in adults.

Overall, our results tentatively suggest that durational cues are more relevant for speech segmentation than pitch cues (cf. Männel and Friederici, 2016), and that boundary cues of pauses and length might play a bigger role for segmentation than language-specific stress patterns, at least for the manipulation sizes employed here.

One possible reason for the primacy of durational information is that durational changes are language-universally more reliable cues for domain-finality than are pitch changes (Vaissière, 1983; Tyler and Cutler, 2009; Fletcher, 2010). In contrast, pitch changes often map onto language-specific word stress patterns (Tyler and Cutler, 2009; Ordin and Nespor, 2013, 2016; Ordin et al., 2017). In real speech, word stress can vary more than domain finality at the phrasal level (Ordin and Nespor, 2016), e.g., due to loan words with non-typical stress patterns (Broselow, 2009; Speyer, 2009; Andersson et al., 2017). Thus, pitch cues in natural speech may be employed more flexibly and variably than durational cues, making them less informative for speech segmentation. Although pitch changes also occur domain finally in real speech (e.g., final pitch decrease in declarative sentences or final pitch increase in yes-no questions; Vaissière, 1983), they may not have the same perceptual salience as durational cues. This may also explain why, overall, we found no clear differences between two opposing pitch changes: pitch decrease and pitch increase.

### Robustness of the Results and Sensitivity to the Testing Environment

Although our results were consistent overall in the three studies, somewhat surprisingly, the effects described above unfolded most clearly in the most informal Experiment 1, less clearly in laboratory Experiment 2 and least clearly in Experiment 3. Experiment 3 was probably closest to most previous artificial language learning experiments in the literature. Thus, despite their overall consistency, our results were sensitive to differences in the experimental environment and the overall testing paradigm (for an overview of the methodological differences between the experiments and a summary of the results, see Table 3). Indeed, only in Experiment 1 did we replicate the finding that statistical cues alone suffice for successful speech segmentation, despite such effects being well-attested in the literature (e.g., Saffran et al., 1996a; Aslin et al., 1998).

**Table 3:** Summary of the methodological details and main results of the three experiments.

	**Methods**				**Main results**			
	**Setting**	**Design**	**Modality of test stimuli**	**Language of stimuli speaker**	**Baseline**	**Pauses**	**Durational cues**	**Pitch cues**
Exp. 1	Natural	Between-subjects	Visual	English	Successful segmentation	Improve segmentation	Lengthening improves & shortening hinders segmentation	No effect compared to baseline
Exp. 2	Lab	Within-subjects	Visual	German	No successful segmentation	Improve segmentation	Lengthening: successful segmentation, no improvement compared to baseline Shortening hinders segmentation	Not tested
Exp. 3	Lab	Within-subjects	Auditory	German	No successful segmentation	Improve segmentation	No effect compared to baseline; tendency: lengthening improves & shortening hinders segmentation	No effect compared to baseline; tendency: higher pitch improves & lower pitch hinders segmentation

A potential explanation for why the effects unfolded most clearly in Experiment 1 might be that in Experiments 2 and 3, in which participants were tested on more than one condition, participants were less focused in later stages of the experiment, which may be reflected in their overall segmentation scores. Also, in Experiments 2 and 3, participants may have inferred a segmentation rule (such as “the modified syllable is always the initial/medial/final syllable of the word”) early on that they then transferred to later conditions. Depending on the rule they formed, this could either facilitate or impair segmentation in subsequent conditions. Because of the randomized order of the conditions and the null effect of the factor *order of the conditions* in our models, it is unlikely that there were consistent biases in a specific direction, but overall, cross-condition interference may have led to fuzzier results in Experiments 2 and 3.

The fact that Experiment 1 used syllables recorded by a native speaker of English does not seem to have influenced the overall pattern of results. If sub-phonemic cues in the English syllables had confused the listeners, results in Experiment 1 would have been expected to be fuzzier. In contrast, listeners may even have applied language-universal segmentation strategies such as final lengthening more consistently in Experiment 1 because they may have recognized that the syllables were not German and in turn reasoned that German-specific segmentation strategies may not be reliable in this case (cf. Quam and Creel, 2017).

### Response Strategies in the Three Different Experiments

The slightly different setups in the three experiments appear to have led to different response strategies of the participants. In Experiment 2, participants made their choices most conservatively, meaning that overall they selected fewer test items as “words.” This led to many misses of words and in general a lower performance on the identification of statistical words than on the rejection of statistical part-words. One potential reason is that, when participants tentatively identified a word, they then waited until this word reoccurred in the speech stream before confirming their choice and circling the item on the test sheet. They might not have had adequate time using this conservative strategy to identify all words while the speech stream was playing. However, participants did not exhibit this behavior in Experiment 1, although there they only had half the stimulus exposure as in Experiment 2. It is also possible that in the laboratory environment in Experiment 2, participants used explicit learning mechanisms, were more nervous or more concerned about doing well in the task and therefore answered more carefully and conservatively (cf. Parsons, 1974; Wickstrom and Bendix, 2000; Chiesa and Hobbs, 2008), whereas the informal environment in Experiment 1 triggered more implicit learning mechanisms, and elicited more immediate and thus perhaps more natural and spontaneous responses.

In contrast to Experiment 2, Experiment 3 was also in the laboratory, but this time had a pronounced auditory memory component. Here, participants overall chose very many items as “words.” This led to many false alarms and in general a poor performance on statistical part-words compared to statistical words. Potentially, participants may have distrusted their memory and selected many items that sounded similar to those that they remembered. Overall, the differences between these two experiments suggests that when the task involves a pronounced memory component (similar to real language learning), speech segmentation becomes more challenging. On the one hand, the additional cognitive load of having to remember some segmented words might have made it more challenging for participants to extract later items from the stream. On the other hand, participants might have segmented many words correctly while listening to the speech stream, but then forgotten them later during the test phase. In any case, although participants performed worse in Experiment 3, their overall response patterns differed between the different cues as in the previous two experiments. The slightly different response patterns observed in our three experiments suggest that future speech segmentation studies should pay careful attention to such seemingly minor experimental differences, and it may be valuable to increase ecological validity by designing tasks that resemble real-life language learning more closely.

## Conclusion and Outlook

In sum, our study provides new insights into how different prosodic cues aid or hinder statistics-based speech segmentation in native German-speaking adults. Because our study only manipulated word-final syllables, it would be interesting to replicate our study using the same manipulations, but on the initial or medial syllables of trisyllabic words (as done in Saffran et al., 1996b; Toro-Soto et al., 2007; Toro et al., 2009; Tyler and Cutler, 2009; Ordin and Nespor, 2016; Frost et al., 2017; Ordin et al., 2017; but these studies did not test opposing cues in direct comparison). Such a research program would provide a more comprehensive overview of the influence of the different individual cues in different locations. Our results make clear predictions for follow up-experiments with cues implemented on the medial syllables, especially for shortening cues. Since our study indicates that shortening word-medially sounds “most natural,” even when this conflicts with statistical cues, medial shortening cues that match the statistical cues should lead to a higher segmentation performance (at least for German speakers). On the contrary, medial lengthening should hinder statistics-based segmentation performance.

Further tests manipulating word-initial cues would also be interesting with regard to the iambic-trochaic law (= ITL; Bolton, 1894; Hayes, 1995; Hay and Saffran, 2012; De la Mora et al., 2013; Abboub et al., 2016). Our study provides further evidence that, considering durational cues, the ITL generalizes from disyllabic to trisyllabic stimuli, namely that lengthened syllables are interpreted as word-final and lead to anapestic grouping (cf. Saffran et al., 1996b; Trainor and Adams, 2000; Tyler and Cutler, 2009; Frost et al., 2017). However, our null results regarding pitch modifications did not provide clear evidence regarding whether the ITL also generalizes to trisyllabic stimuli, leading to dactylic grouping. According to the ITL, higher pitched syllables are grouped sequence initially, so we predicted for our study that final pitch increase should hinder speech segmentation performance. However, this was not evident in our data. Thus, a variant of our experiment manipulating word-initial pitch would test more directly whether the ITL also transfers to trisyllabic stimuli for pitch modifications. If initial pitch increase indeed turned out to lead to dactylic grouping, this, combined with our finding about anapestic grouping of lengthened syllables, would point toward an “anapest-dactyl law” for trisyllabic stimuli, directly analogous to the ITL for bisyllabic stimuli.

Overall, we showed that different prosodic cues, namely pauses after the final syllables of trisyllabic statistical words, and durational and pitch cues on the final syllables of such words, had differing effects on speech segmentation. More specifically, pauses were most salient, duration changes also significant, and pitch changes showed little or no effect. Our findings are consistent with previous results indicating that when in conflict, prosodic cues can override statistical cues (e.g., Johnson and Jusczyk, 2001; Thiessen and Saffran, 2003; Johnson and Seidl, 2009). In addition, we found that changes in a single prosodic cue—duration—were enough to achieve such an override. Because we tested opposing cues—lengthening vs. shortening and pitch increase vs. decrease—in direct comparison, we were able to show that overall, durational cues played a more important and consistent role than pitch cues. These results contribute to a better understanding of which specific acoustic factors are most salient for listeners as they solve the challenge of speech segmentation.

Like most previous experimental work, our study tested speech segmentation in an artificial language with highly controlled and simplified stimuli and cue manipulations (although our study did use modified natural speech, rather than synthesized speech). This control and simplification has the virtue that the effects can be attributed to specific individual cues, but also raises the problem of how well these findings will translate to the segmentation of natural languages, where cues hardly ever occur in isolation and are more complex (Johnson and Seidl, 2009; Johnson and Tyler, 2010; Erickson and Thiessen, 2015). Although the full complexity of natural languages is hard to model in speech segmentation experiments in a controlled way, one step toward natural language conditions is to test durational, pitch and pause cues in combination, either converging or conflicting (like Ordin and Nespor, 2016 did for lengthening and pitch increase cues), but additionally adding pause, shortening and pitch decrease cues. This can shed light on whether the effects of cue changes are simply additive or if they interact in more complex ways when occurring in combination. Further factors that could move this research field toward natural languages include adding cues such as co-articulation cues (as e.g., in Johnson and Jusczyk, 2001), adding cues distinguishing between different boundary strengths (as e.g., in Sohail and Johnson, 2016), modifying the surrounding prosodic context (Morrill et al., 2014a,b, 2015), using words of different lengths and syllable structures (as e.g., in Johnson and Tyler, 2010), or incorporating prior lexical knowledge (as e.g., in Mattys et al., 2005), cues about syntactic structure (as e.g., Mueller et al., 2018), or even visual facial expression cues (as e.g., in Mitchel and Weiss, 2014). These all could be integrated in segmentation experiments with contrasting lengthening and pitch cues.

## Data Availability Statement

The datasets presented in this study can be found in online repositories. The names of the repository/repositories and accession number(s) can be found here: https://osf.io/xtf6k/.

## Ethics Statement

The studies involving human participants were reviewed and approved by the ethics board of the University of Vienna. The patients/participants provided their written informed consent to participate in this study.

## Author Contributions

TM: conceptualization, methodology, software, investigation, and writing—original draft preparation. NR: supervision and writing—review & editing. WTF: conceptualization, methodology, supervision, and writing—review & editing. All authors contributed to the article and approved the submitted version.

## Conflict of Interest

The authors declare that the research was conducted in the absence of any commercial or financial relationships that could be construed as a potential conflict of interest.
