# Supplementary material for: The Influence of Different Prosodic Cues on Word Segmentation
Source: Front Psychol. 2021 Mar 16;12:622042. doi: 10.3389/fpsyg.2021.622042 (PMC8007974; doi:10.3389/fpsyg.2021.622042)
Supplement: Supplementary file 1 [file Data_Sheet_1.PDF]

The influence of different prosodic cues on word segmentation

Theresa Matzinger[1,2*], Nikolaus Ritt[1], W. Tecumseh Fitch[2,3*]

[1] Department of English, University of Vienna, Spitalgasse 2/Hof 8, 1090 Vienna;

[2] Department of Behavioral and Cognitive Biology, University of Vienna, Althanstraße 14, 1090 Vienna;

[3] Cognitive Science Hub, University of Vienna, Vienna, Austria.

Supplementary materials

Experiment 1

Table S1. Results of the logistic generalized mixed model exploring the effects of condition, stimulus type, and their interaction, on the correctness of responses in a decision task where participants had to decide on whether presented stimuli were words or not. The table reports estimated model coefficients, standard errors (SE), lower and upper confidence intervals for the estimates, χ^2-values of likelihood ratio tests, respective degrees of freedom (df) and p-values (P, significances in bold), as well as minimum and maximum estimates obtained after dropping individuals one at a time (an indicator of model stability).

Term	Estimate	SE	Lower CI	Upper CI	χ^2	df	P	Min. estimate	Max. estimate
Intercept	0.84	0.32	0.23	1.55	(1)	(1)	(1)	0.77	0.94
conditionPAU	2.87	0.62	1.76	4.33				2.74	3.30
conditionLEN	0.94	0.48	-0.01	1.86				0.82	1.10
conditionSHO	-2.83	0.49	-3.87	-1.87				-2.93	-2.76
conditionLOW	0.46	0.46	-0.49	1.41				0.35	0.59
conditionHIG	0.25	0.46	-0.71	1.18				0.13	0.37
partword_typePW1	0.13	0.40	-0.68	0.95				0.00	0.27
partword_typePW2	-0.56	0.32	-1.20	0.06				-0.66	-0.42
conditionPAU:partword_typePW1	-1.46	0.73	-3.14	0.04	63.129	10	**<0.001**	-1.84	-1.20
conditionLEN:partword_typePW1	0.27	0.61	-0.97	1.45				0.12	0.45
conditionSHO:partword_typePW1	3.31	0.60	2.08	4.63				3.16	3.53
conditionLOW:partword_typePW1	0.27	0.58	-0.92	1.45				0.10	0.48
conditionHIG:partword_typePW1	0.18	0.57	-1.04	1.31				0.04	0.39
conditionPAU:partword_typePW2	-0.64	0.66	-2.18	0.64				-0.94	-0.40
conditionLEN:partword_typePW2	1.34	0.55	0.25	2.51				1.14	1.57
conditionSHO:partword_typePW2	2.32	0.50	1.40	3.40				2.17	2.47

conditionLOW:partword_typePW2	0.14	0.47	-0.81	1.00				0.00	0.29
conditionHIG:partword_typePW2	0.32	0.47	-0.65	1.26				0.18	0.45

(1) Not shown because it allows very limited interpretation

Experiment 2

Table S2. Results of the logistic generalized mixed model exploring the effects of condition, stimulus type, their interaction, order of the conditions, and language on the correctness of responses in a decision task where participants had to decide on whether presented stimuli were words or not. The table reports estimated model coefficients, standard errors (SE), lower and upper confidence intervals for the estimates, χ^2-values of likelihood ratio tests, respective degrees of freedom (df) and p-values (P, near-significances in bold), as well as minimum and maximum estimates obtained when dropping individuals one at a time (being an indicator of model stability).

**Term**	**Estimate**	**SE**	**Lower CI**	**Upper CI**	χ^2	**df**	**P**	**Min. estimate**	**Max. estimate**
Intercept	-0.45	0.31	-1.05	0.15	(1)	(1)	(1)	-0.55	-0.35
conditionPAU	2.24	0.43	1.45	3.22				2.07	2.42
conditionLEN	1.12	0.34	0.50	1.84				1.01	1.29
conditionSHO	-0.77	0.32	-1.39	-0.18				-0.92	-0.61
partword_typePW1	1.69	0.40	0.94	2.52				1.55	1.90
partword_typePW2	1.42	0.42	0.63	2.27				1.27	1.62
z.condition_order_no (2)	-0.09	0.10	-0.27	0.10	0.954	1	0.329	-0.14	-0.06
languageLanguage2 (3)	-0.09	0.24	-0.55	0.39	1.725	3	0.631	-0.22	0.00
languageLanguage3	-0.10	0.24	-0.56	0.39				-0.21	0.04
languageLanguage4	0.17	0.25	-0.31	0.71				0.06	0.29
conditionPAU:partword_typePW1	-1.02	0.49	-2.00	-0.05	11.329	6	**0.079**	-1.21	-0.78
conditionLEN:partword_typePW1	-0.76	0.46	-1.66	0.17				-0.92	-0.61
conditionSHO:partword_typePW1	0.30	0.53	-0.72	1.30				0.05	0.50
conditionPAU:partword_typePW2	-1.07	0.47	-2.07	-0.16				-1.26	-0.87
conditionLEN:partword_typePW2	-0.31	0.49	-1.28	0.73				-0.61	-0.15
conditionSHO:partword_typePW2	0.16	0.42	-0.68	0.97				0.02	0.30

(1) Not shown because it allows very limited interpretation
(2) z-transformed mean and standard deviation of the original variable were 1.50 and 1.12 respectively
(3) *language* was manually dummy coded with Language1 being the reference category, and then centered

Experiment 3

Table S3. Results of the logistic generalized mixed model exploring the effects of condition, stimulus type, their interaction, order of the conditions, trial number, and language on the correctness of responses in a decision task where participants had to decide on whether presented stimuli were words or not. The table reports estimated model coefficients, standard errors (SE), lower and upper confidence intervals for the estimates, χ^2-values of likelihood ratio tests, respective degrees of freedom (df) and p-values (P, significances in bold), as well as minimum and maximum estimates obtained when dropping individuals one at a time (being an indicator of model stability).

**Term**	**Estimate**	**SE**	**Lower CI**	**Upper CI**	χ^2	**df**	**P**	**Min. estimate**	**Max. estimate**
Intercept	0.97	0.19	0.60	1.39	(1)	(1)	(1)	0.92	1.06
conditionPAU	1.29	0.34	0.61	1.95				1.18	1.45
conditionLEN	0.30	0.38	-0.39	1.08				0.16	0.43
conditionSHO	-0.87	0.33	-1.50	-0.21				-1.01	-0.71
conditionLOW	-1.28	0.33	-1.94	-0.68				-1.39	-1.14
conditionHIG	0.00	0.32	-0.60	0.63				-0.16	0.20
partword_typePW1	-1.79	0.27	-2.38	-1.31				-1.93	-1.70
partword_typePW2	-1.52	0.25	-2.02	-1.06				-1.68	-1.43
z.condition_order_no (2)	0.02	0.06	-0.10	0.15	0.159	1	0.692	-0.01	0.04
z.trial_no (3)	-0.01	0.05	-0.11	0.10	0.014	1	0.907	-0.02	0.02
languageLanguage2 (4)	-0.12	0.18	-0.48	0.22	4.013	3	0.260	-0.19	-0.07
languageLanguage3	0.20	0.18	-0.14	0.58				0.08	0.29
languageLanguage4	0.15	0.17	-0.19	0.48				0.07	0.22
conditionPAU:partword_typePW1	-0.15	0.40	-0.99	0.68	31.963	10	**< 0.001**	-0.26	0.03
conditionLEN:partword_typePW1	-0.51	0.46	-1.54	0.33				-0.70	-0.35
conditionSHO:partword_typePW1	1.61	0.56	0.54	2.71				1.33	1.87
conditionLOW:partword_typePW1	1.85	0.47	0.89	2.79				1.67	2.01
conditionHIG:partword_typePW1	0.57	0.47	-0.33	1.50				0.33	0.76
conditionPAU:partword_typePW2	-0.38	0.39	-1.13	0.33				-0.51	-0.19
conditionLEN:partword_typePW2	-0.31	0.44	-1.20	0.53				-0.49	-0.16
conditionSHO:partword_typePW2	0.61	0.44	-0.31	1.47				0.41	0.80
conditionLOW:partword_typePW2	1.67	0.55	0.60	2.78				1.44	1.91
conditionHIG:partword_typePW2	0.51	0.42	-0.27	1.34				0.17	0.75

(1) Not shown because it allows very limited interpretation

(2) z-transformed, mean and sd of the original variable were 1.50 and 1.12, respectively
(3) z-transformed, mean and sd of the original variable were 5.50 and 3.45, respectively
(4) *language* was manually dummy coded with Language1 being the reference category, and then centered
